# Evaluation of behavioural selection processes in conflict scenarios using a newly developed mouse behavioural paradigm

**DOI:** 10.1038/s41598-023-46743-3

**Published:** 2023-11-16

**Authors:** Yurika Miyagami, Yuki Honshuku, Hiroshi Nomura, Masabumi Minami, Natsuko Hitora-Imamura

**Affiliations:** 1 Department of Pharmacology, Graduate School of Pharmaceutical Sciences, Hokkaido University, Sapporo, 060-0812 Japan; 2 Department of Chemico-Pharmacological Sciences, Graduate School of Pharmaceutical Sciences, Kumamoto University, Kumamoto, 862-0973 Japan; 3 Department of Cognitive Function & Pathology, Institute of Brain Science, Nagoya City University Graduate School of Medical Sciences, Nagoya, 467-8601 Japan

**Keywords:** Emotion, Learning and memory, Motivation, Reward

## Abstract

Selecting an appropriate behaviour is critical for survival in conflict scenarios, wherein animals face both appetitive and aversive stimuli. Behavioural selection consists of multiple processes: (1) animals remain quiet in a safe place to avoid aversive stimuli (suspension), (2) once they decide to take risks to approach appetitive stimuli, they assess the risks (risk assessment), and (3) they act to reach the reward. However, most studies have not addressed these distinct behavioural processes separately. Here, we developed a new experimental paradigm called the three-compartment conflict task to quantitatively evaluate conflict processes. Our apparatus consisted of start, flat, and grid compartments. Mice needed to explore the grid compartment, where they might receive foot shocks while trying to obtain sucrose. Applying foot shocks increased sucrose acquisition latency in subsequent trials, reflecting elevated conflict levels throughout trials. The time spent in the start compartment and the number of retreats were determined to measure the conflict levels in suspension and risk assessment, respectively. Foot shocks increased these parameters, whereas diazepam decreased them. Our new paradigm is valuable for quantitatively evaluating distinct behavioural processes and contributes to developing effective treatments for psychiatric disorders associated with maladaptive behaviours in conflict scenarios.

## Introduction

We are constantly surrounded by a range of appetitive and aversive stimuli. Appetitive stimuli encourage us to engage in certain behaviours, such as the smell of food, leading to food-seeking behaviours. In contrast, aversive stimuli prevent us from displaying certain behaviours or induce escape behaviours. The sight of a predator or cues associated with painful experiences induces freeze-or-flight behaviour. However, a mixture of appetitive and aversive stimuli makes it difficult to select the appropriate behaviour, as animals make behavioural decisions in an approach-avoidance conflict scenario.

Animals in conflict situations face two questioning steps before reaching a reward^1^. The first is whether to act; that is, to approach and retrieve the reward. Until they reach a decision, they remain in a safe place (suspension). If they decide positively in the first step, they must then determine when and how to act. To decide their course of action in the second step, they assess the risks at a place where danger might be present (risk assessment) to finally reach the reward. Studying these distinct behavioural processes is important because suspension and/or risk assessment abnormalities have been observed in several psychiatric disorders. For example, patients with attention deficit hyperactivity disorder (ADHD) and drug abusers have difficulty suspending a response in the face of negative consequences^2–4^. Moreover, patients with anxiety disorders often overestimate their risk assessment of ambiguous threat information^5–7^.

Conflict behaviour tests have long been used in rodents to evaluate the effect of anxiolytic drugs^8,9^. An operant conflict test is a good example of a commonly used animal model. Animals learn to associate a rewarded action, such as drinking water in the Vogel task^10^ or pressing a lever for a food reward in the Geller–Seifter task^11^, with a punishment, such as a mild electric shock. The maintenance of the rewarded action despite receiving the shock is considered an anxiolytic-like behaviour. However, these tasks are not appropriate for evaluating suspension and risk assessment because they focus on evaluating the final decision and not on monitoring the processes leading to the decision.

In the present study, we developed a new paradigm, a three-compartment conflict task, which enables the quantitative evaluation of distinct behavioural processes in mice. We confirmed the pharmacological validity of the task using diazepam (DZP), which has been reported to have an anti-conflict effect^12–15^.

## Results

### Foot shocks increased suspension and risk assessment behaviour in a three-compartment conflict task

**Figure 1:**
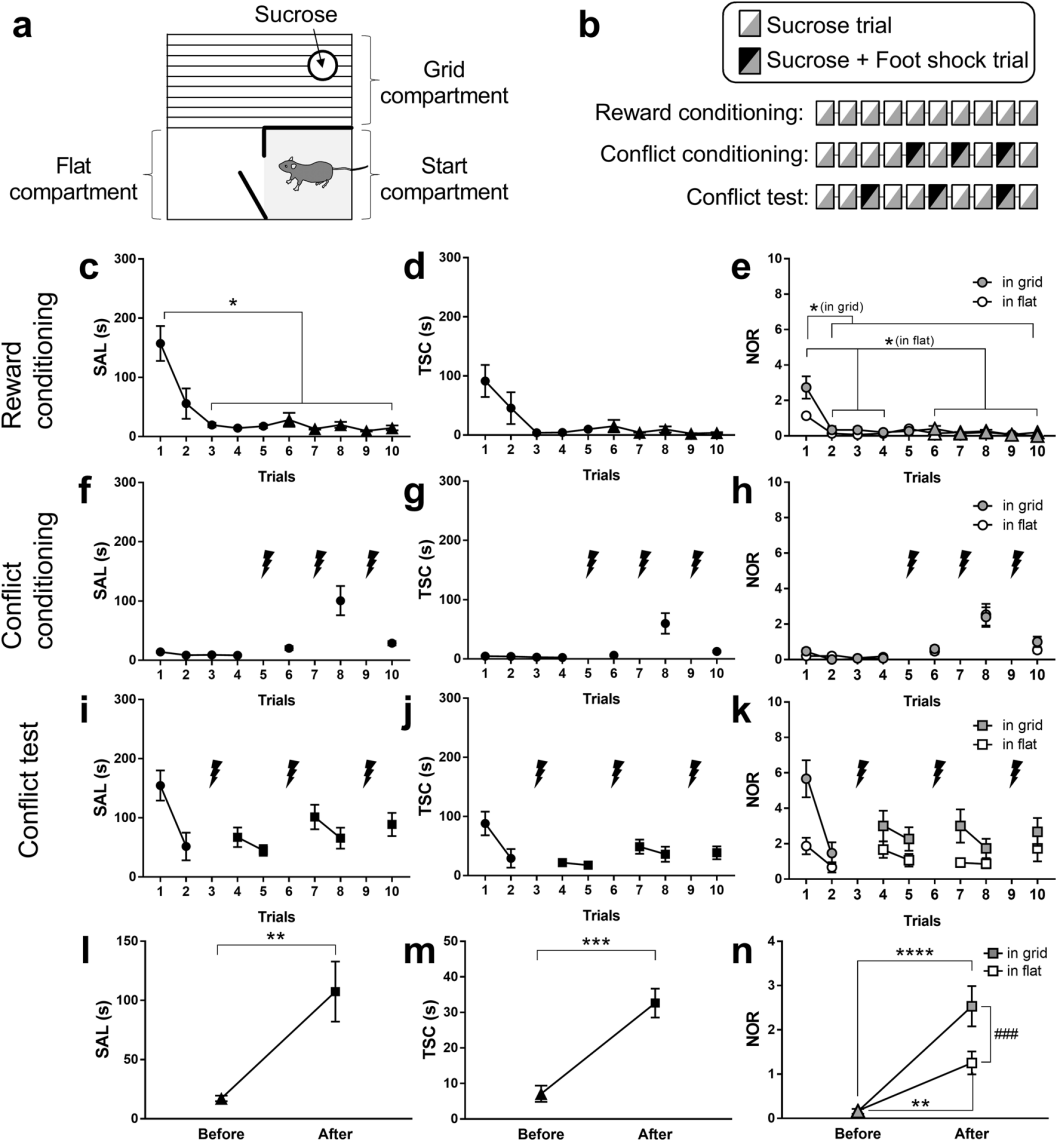
Basal performance in the three-compartment conflict task. (**a**) Experimental apparatus. (**b**) Conditioning and testing procedures. (**c**) Sucrose acquisition latency (SAL) during reward conditioning. One-way RM-ANOVA, F = 12.86, *P* < 0.0001; Tukey’s multiple comparison test, *P < 0.05. (**d**) Time spent in the start compartment (TSC) during reward conditioning. (**e**) Number of retreats (NOR) during reward conditioning. Two-way RM-ANOVA, F_9,126_ = 11.76, *P* < 0.0001; Sidak’s multiple comparison test, *P < 0.05. (**f**–**h**) Performance during conflict conditioning. (**i**–**k**) Performance during the conflict test. Lighting symbols () indicate the trials with foot shocks. (**l**–**n**) Comparison of the performance before (triangles; trials 6–10 of the reward conditioning) and after (squares; trials 4, 5, 7, 8, and 10 of the conflict test) conflict conditioning. Paired *t*-test; SAL: t_(14)_ = 3.45, *P* = 0.0039; TSC: t_(14)_ = 4.69, *P* = 0.0003. NOR: Two-way RM-ANOVA followed by Sidak’s multiple comparisons test; F_time 1,14_ = 28.65, *P* = 0.0001, *P*_flat_ = 0.0012, *P*_grid_ < 0.0001; F_place 1,14_ = 12.62, *P* = 0.0032. *P*_after_ = 0.0003. Before versus after: ****P < 0.0001, ***P < 0.001, **P < 0.01. Flat versus grid: ^###^*P* < 0.001; n = 15. Data represent the mean ± standard error. For details of statistical comparisons, see the Supplementary Information.

In the three-compartment conflict task, mice could obtain a sucrose solution in the grid compartment of a behavioural apparatus, where they may receive a foot shock. The mice began the tasks from the start compartment of the apparatus and could reach the grid compartment through the flat compartment (Fig. 1a). The latency to obtain sucrose (sucrose acquisition latency, SAL) was measured as a parameter of conflict levels throughout the trials. The time spent in the start compartment (TSC) and the number of retreats (NOR) were determined to measure the levels of conflict in suspension and in risk assessment, respectively. First, mice were trained to obtain sucrose on a plate at the end of the grid compartment (reward conditioning; Fig. 1b). The SAL and NOR decreased as reward conditioning progressed (Fig. 1c–e). The next day, the mice received a weak immediate foot shock (0.1 mA, 0.5 s) when they first stepped on the grid compartment in trials 5, 7, and 9 (conflict conditioning; Fig. 1b,f–h). The next day, the subjects underwent conflict tests, in which they received a foot shock in trials 3, 6, and 9 (Fig. 1b,i–k). To check the effect of conflict conditioning on these parameters, data after conflict conditioning (trials 4, 5, 7, 8, and 10 of the conflict test) were compared with data before conditioning (trials 6–10 of reward conditioning). The SAL, TSC, and NOR values increased after conflict conditioning compared with that before conditioning (Fig. 1l–n). The NOR in the grid compartment was higher than those in the flat compartment (Fig. 1n).

### Foot shock intensity affects conflict behaviour

**Figure 2:**
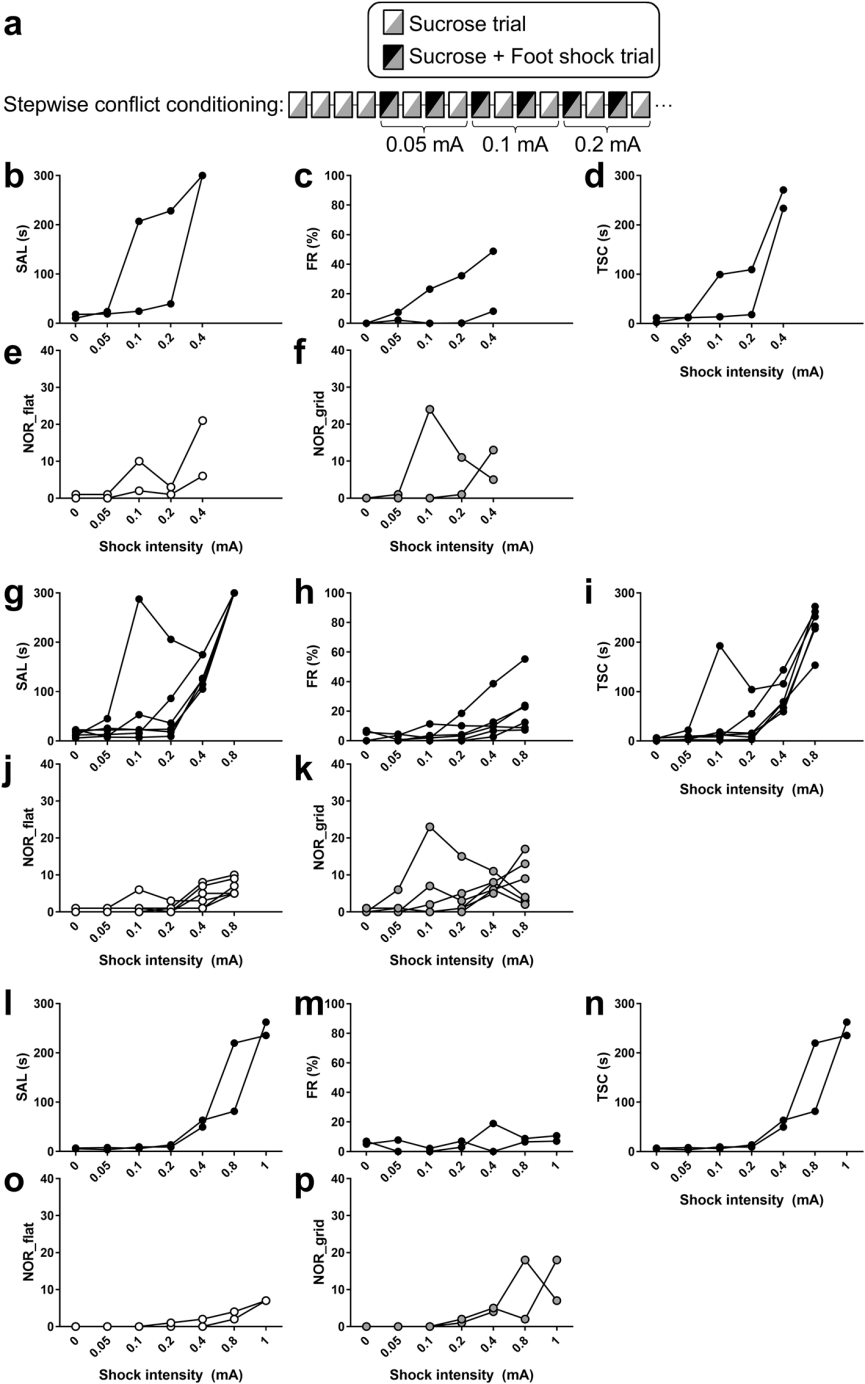
Performance of individual mice in the stepwise conflict test. (**a**) Stepwise conflict test procedure. (**b**–**f**) Performance of individual mice at maximum intensities of up to 0.4 mA; n = 2. (**g**–**k**) Performance of individual mice at maximum intensities of up to 0.8 mA; n = 6. (**l**–**p**) Performance of individual mice at maximum intensities of up to 1 mA; n = 2.

**Figure 3:**
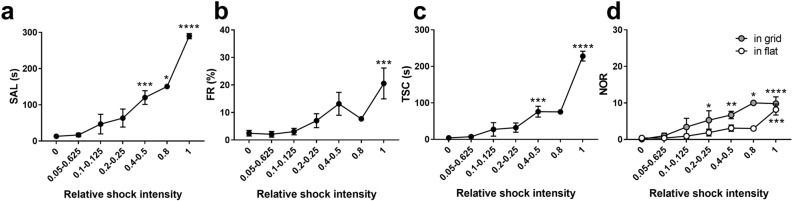
Effect of foot shock intensity on conflict behaviour. (**a**) Average sucrose acquisition latency (SAL) aligned with shock intensity relative to the maximum intensity. One-way ANOVA, F_6,53_ = 28.08, *P* < 0.0001; Dunnett’s multiple comparisons test, ****P < 0.0001, ***P < 0.001, *P < 0.05. (**b**) Average freezing rate (FR) aligned with shock intensity relative to the maximum intensity. One-way ANOVA, F_6,53_ = 4.513, *P* = 0.0009; Dunnett’s multiple comparison test, ***P < 0.001. (**c**) Average time spent in the start compartment (TSC) aligned with shock intensity relative to the maximum intensity. One-way ANOVA, F_6,53_ = 40.95, *P* < 0.0001; Dunnett’s multiple comparison test, ****P < 0.0001, ***P < 0.001. (**d**) Average number of retreats (NOR) aligned with shock intensity relative to the maximum intensity. Two-way RM-ANOVA, F_6,53_ = 7.335, *P* < 0.0001; Dunnett’s multiple comparisons test, ****P < 0.0001, ***P < 0.001, **P < 0.01, *P < 0.05; n = 10. Data represent the mean ± standard error.

To validate whether the three-compartment conflict task was sensitive to the conflict level, we manipulated the shock intensity. Ten mice were subjected to conflict conditioning in a stepwise manner in which the strength of the shock intensity was progressively increased until they gave up obtaining a reward (Fig. 2a). Among the ten mice, the maximum intensities were 0.4 mA (Fig. 2b–f) for two mice, 0.8 mA (Fig. 2g–k) for six mice and 1.0 mA (Fig. 2l–p) for two mice. Because the maximum intensity for each mouse was variable, the meaning of the absolute value of the shock intensity may have differed for each animal. Therefore, we averaged the results of the relative shock intensities (shock intensity/maximum intensity) for the ten mice. The SAL increased as shocks increased in strength (Fig. 3a), accompanied by an increase in the freezing rate (FR; Fig. 3b), a parameter of fear response^16,17^. The TSC (Fig. 3c) and NOR in the grid compartment (Fig. 3d) also increased as the shocks became stronger, suggesting a progressive increase in conflict levels in both suspension and risk assessment. The NOR in the grid compartment increased between “0.2” and “1” of the relative shock intensity. In contrast, the NOR in the flat compartment rose sharply at “1” of the relative shock intensity, indicating that, at the maximum intensity, fear generalises to the flat compartment where they never received any foot shocks (Fig. 3d).

### The anti-conflict effect of DZP was demonstrated in the three-compartment conflict task

**Figure 4:**
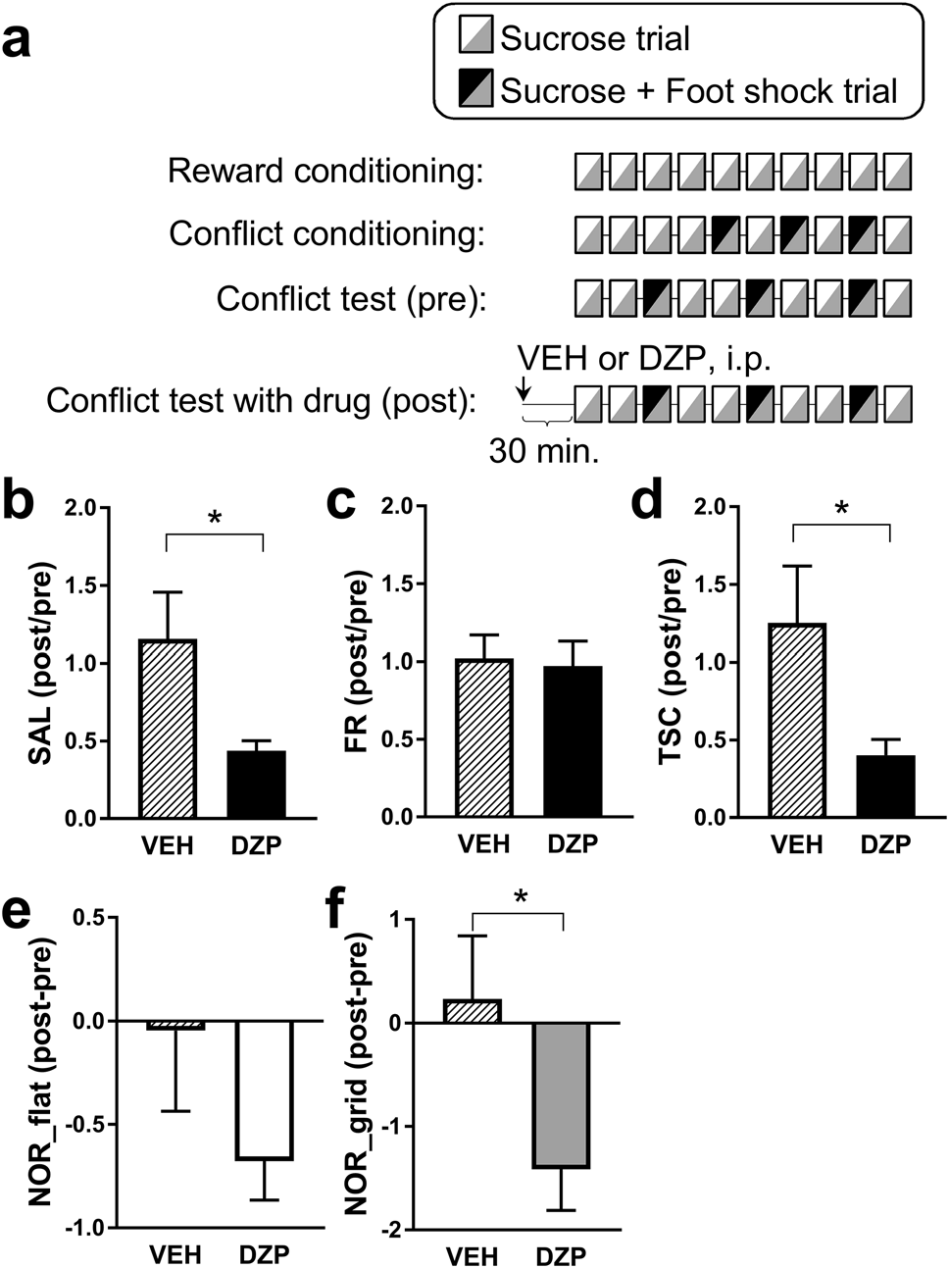
Effect of diazepam on conflict behaviour. (**a**) Setup of the pharmacological experiments. (**b**–**f**) Effects of diazepam (DZP, 1.0 mg/kg, i.p.) on the sucrose acquisition latency (SAL), freezing rate (FR), time spent in the start compartment (TSC), and number of retreats (NOR) values; n = 13. Student’s *t*-test; SAL: t _(24)_ = 2.323, *P* = 0.0290; FR: t _(24)_ = 0.235, *P* = 0.8162; TSC: t _(24)_ = 2.230, *P* = 0.0354; NOR_flat: t _(24)_ = 1.455, *P* = 0.1587; NOR_grid: t _(24)_ = 2.264, *P* = 0.0329. *P < 0.05. Data represent the mean ± standard error.

Mice were subjected to a conflict test 30 min after intraperitoneal (i.p.) injection of either DZP (1 mg/kg) or vehicle solution (VEH) (“post”), and conflict behaviours were compared to those of the previous day (“pre”; Fig. 4a). DZP demonstrated its anti-conflict effect by decreasing the SAL, TSC, and NOR in the grid compartment but had no effect on FR (Fig. 4b–f). These results demonstrate the anti-conflict effect of DZP in the three-compartment conflict task.

## Discussion

We established a three-compartment conflict task to quantitatively evaluate distinct behavioural processes (suspension and risk assessment) within an approach-avoidance conflict scenario involving both appetitive and aversive stimuli. We demonstrated that applying the optimised shock intensity increased the SAL, TSC, and NOR. Upon experiencing strong shocks, mice retreated not only in the grid compartment but also in the flat compartment, reflecting fear generalisation. We used DZP to assess the pharmacological validity of the three-compartment conflict task and observed that treatment with DZP decreased the SAL, TSC, and NOR. Our results demonstrate the efficacy of a three-compartment conflict task for monitoring behavioural selection processes in mice.

We aimed to quantify distinct behavioural selection processes in conflict scenarios, which can be difficult with existing task protocols such as the Vogel task, Geller–Seifter task, and Pavlovian conditioning tasks in which cues are associated with both reward and shock^10,11,18,19^. In these existing conflict tests, the focus lies on counting rewarded behaviours despite the presence of shocks while often overlooking the preceding processes of suspension and risk assessment. Evaluation of suspension and risk assessment in those tasks is challenging because animals usually remain near a water bottle, lever, or nose-poke hole and occasionally engage in minimal movements to lick, press the lever, or nose-poke. So, it is hard to behaviourally dissociate two decision-making processes—whether to act and when and how to act. To differentiate these two decision-making processes temporally, spatially, and behaviourally, we used an experimental environment consisting of three compartments with different degrees of safety: the safest “start compartment,” the next safest “flat compartment,” which is in close proximity to the grid compartment but where mice had never experienced foot shock, and the highest risk “grid compartment” where mice had experienced foot shock. “Whether to act” occurs in the safest start compartment, and if mice decide to act, they leave the start compartment. This allows us to quantify suspension, which is the process before animals reach the decision of “whether to act,” using the TSC as an indicator. Then, “when and how to act” occurs at the flat and grid compartment. Risk assessment can be quantified by counting NOR at these compartments.

Our task presented punishment and reward consecutively, whereas many previous studies presented reward and punishment simultaneously. The process of first assessing risk at a place where danger might be present and then obtaining a reward if the animal does not encounter aversive stimuli is close to conditions occurring in nature and is suitable for studying the decision-making process in conflict environments. Besides, in order to investigate the neural mechanisms underlying behavioural selection, it is useful to temporally separate appetitive and aversive stimuli, as this allows for distinguishing neural activity in response to each stimulus. In future research, by employing techniques such as calcium imaging and optogenetics, observing and manipulating neural activity related to reward and punishment separately will shed light on how the brain processes these stimuli and the decision-making process under conflict scenarios.

We observed fear generalisation by monitoring the location of retreats. During the relatively weak shocks in the stepwise conflict test, retreats were observed mainly in the grid compartment. However, at the maximum shock intensity, the NOR in the flat compartment surged, indicating a generalisation of fear in the flat compartment. For adaptive behaviour, risk should be assessed in the grid compartment, that is, where the mice had experienced foot shock. On the other hand, elevated risk assessment in the flat compartment, i.e., where they never had experienced foot shock, prevented them from behaving and resulted in a delay or failure to obtain sucrose. Fear generalisation prevents patients with anxiety disorders from behaving even in safe situations, which can strain their daily lives^7,20^. The three-compartment conflict test will be helpful for studying fear generalisation and inadequate risk assessment in conflict situations.

We confirmed the pharmacological validity of the three-compartment conflict task using DZP. Treatment with DZP (1.0 mg/kg) showed an anti-conflict effect by decreasing the SAL, TSC, and NOR values. Although DZP administered at moderately high doses (2.5–5.0 mg/kg) has been reported to abolish fear memory retrieval^21–24^, we found that DZP treatment (1.0 mg/kg) did not affect the percentage of freezing in the three-compartment conflict test. This suggests that fear memory retrieval was intact under the experimental conditions. The neural mechanisms underlying this anti-conflict effect in three-compartment tasks remain unclear. Previous studies using other conflict tasks have proposed an anti-conflict effect of DZP and other benzodiazepine agonists via local injection into the amygdala, dorsal hippocampus, hypothalamus, and dorsal raphe^12,25–27^. These brain regions may also be involved in the anti-conflict effect of DZP observed in the three-compartment conflict task.

Maladaptive behaviour in conflict situations has been observed in several psychiatric disorders. The inability to suspend behaviour despite harmful consequences is well-characterised in drug addiction, ADHD, and obsessive–compulsive disorder^2–4,28^. Overestimation in the risk assessment of ambiguous threat information is common in anxiety disorders, including specific phobia, generalised anxiety disorder, and post-traumatic stress disorder^7,20^. Our new paradigm is a valuable tool for investigating the neural basis of maladaptive conflict behaviours in rodents.

## Methods

### Animals

Behavioural data were collected from 36 male C57BL/6 J mice (8–14 weeks old; SLC, Shizuoka, Japan). The mice were housed in four group cages (27 cm wide, 16.5 cm deep, and 13 cm high) under standard laboratory conditions (23 ± 1 °C, 12 h light/12 h dark cycle and free access to food and water). Mice were fed a standardised mouse diet (LabDiet 5053). Bedding (Paper Clean™, Japan SLC, Shizuoka, Japan), bottles, and water were autoclaved before use. Mice were handled daily for 3 days before behavioural procedures. They were housed individually in a cage (24 cm wide, 11 cm deep, and 13 cm high) from 1 week prior to the behavioural tests until the end of the behavioural tests. Food was restricted (1.5–4.0 g/day) from the day before the start of the conflict behaviour test until the end of the behavioural tests. All experiments were approved by the institutional animal care and use committees of Hokkaido University (approval number: 16-0043) and Kumamoto University (approval number: A2021-139). These experimental protocols were conducted in accordance with the Fundamental Guidelines for Proper Conduct of Animal Experiments and Related Activities in Academic Research Institutions (Ministry of Education, Culture, Sports, Science and Technology, Notice No. 71 of 2006), the Standards for Breeding and Housing of and Pain Alleviation for Experimental Animals (Ministry of the Environment, Notice No. 88 of 2006), and the Guidelines on the Method of Animal Disposal (Prime Minister's Office, Notice No. 40 of 1995). Animal use followed the recommendations of the ARRIVE guidelines.

### Three-compartment conflict task

A total of 15 mice were used for the experiments shown in Fig. 1. The experimental apparatus (18 cm wide, 15 cm deep, and 27 cm high) consisted of start, flat, and grid compartments (Fig. 1a). The start and flat compartments had a smooth white floor, whereas the grid compartment had a grid floor. The start compartment was enclosed by walls and a door, which separated the start and flat compartments. One day before the behavioural test, the mice were pre-exposed to sucrose in their home cage and were allowed to explore the experimental apparatus for 10 min (habituation). The next day, they were placed in the start compartment until the door opened a minute later. They obtained a 10% sucrose solution when they reached the other end of the grid compartment (sucrose trial). If sucrose was not obtained within 5 min, the trial was terminated. The mice underwent ten sucrose trials (reward conditioning, Fig. 1b). The next day, they underwent four sucrose trials to ensure that reward conditioning was established. Mice continued to the next step only when the average latency of the third and fourth trials was less than 30 s (criterion A). All the tested mice reached criterion A and proceeded to the next step. In the next step, they received a weak immediate foot shock (0.1 mA, 0.5 s) delivered through a shock scrambler (SGS-003DX; Muromachi Kikai, Tokyo, Japan) when they stepped on the grid compartment (sucrose + foot shock trial) in trials 5, 7, and 9. All mice underwent a total of ten trials (conflict conditioning, Fig. 1b). The following day, the mice underwent ten trials, in which they received a foot shock in trials 3, 6, and 9 (conflict test, Fig. 1b). Each trial was video-recorded for automatic analysis using an EthoVision video tracking system (Noldus Information Technology, Wageningen, The Netherlands), custom MATLAB scripts (MathWorks, Natick, MA, USA), and custom Bonsai scripts^29^. The SAL, TSC, NOR, and FR (%) values during the sucrose trials were calculated. The SAL was defined as the time from when the experimenter opened the door until the mouse’s nose rested on the sucrose plate for 2 s. The TSC was the total time the mouse’s centre of gravity was in the start compartment. The retreat was defined as the centre of the mouse head passing from the start compartment to the flat compartment, or from the flat compartment to the grid compartment, stopping forward movement followed by returning until the centre of the head passed between the compartments. Freezing was defined as the absence of movements except those related to breathing^30,31^. Images were captured at 15 frames per second. The area (pixels) within which the mouse moved was measured for each pair of successive frames. When this area was below a threshold, the behaviour was considered “freezing.” The optimal freezing threshold was determined by adjusting to the amount of freezing measured by human observation. In Fig. 1l–n, data from the sixth to tenth trials of the reward conditioning were used as “before the conflict conditioning,” and data from the fourth, fifth, seventh, eighth, and tenth trials of the conflict test were used as “after the conflict conditioning.”

### Stepwise conflict test

Ten mice were used for the experiments depicted in Fig. 2. The mice underwent habituation and reward conditioning, as described above. On the day of the stepwise conflict test, the mice first underwent four sucrose trials to ensure that reward conditioning was established. They proceeded to the next step only when the average latency of the third and fourth trials was less than 30 s. In the next step, they received variable intensities of an immediate 0.5-s foot shock (0.05, 0.1, 0.2, 0.4, 0.8, and 1.0 mA) once in two trials (Fig. 2a). Each session consisted of two sucrose + foot shock trials with the same shock intensity and two intermittent sucrose trials. The shock intensity was 0.05 mA in the first session and was progressively increased. The sessions were terminated when the mice did not obtain sucrose in either sucrose trials or when the shock intensity reached 1.0 mA. The shock intensity used in the last session was the maximum intensity for the mouse. The SAL, TSC, NOR, and FR values in the sucrose trials were calculated. Data from the third and fourth trials were considered 0 mA. In Fig. 3, the shock intensity relative to the maximum intensity for an individual mouse was calculated (shock intensity/maximum intensity) and assigned to one of seven points: 0, 0.05–0.0625, 0.1–0.125, 0.2–0.25, 0.4–0.5, 0.8, or 1.

### Pharmacological experiments

A total of 26 mice were randomly divided into two groups (13 mice/group): the VEH and DZP groups. Fifteen of these mice (8 in VEH group and 7 in DZP group) were the same mice used for the experiments depicted in Fig. 1. DZP (Wako Junyaku, Hokkaido, Japan), an anxiolytic benzodiazepine, was dissolved in saline (0.9% NaCl) mixed with 0.1% Tween-80 (Kanto Chemical, Hokkaido, Japan) and stored at 4 °C for a maximum of 24 h. Saline with 0.1% Tween-80 was used as the VEH solution. Pharmacological manipulation (DZP or VEH injection) was performed after criterion B was met in a conflict test (“pre”). If criterion B was not met, the mice underwent the conflict test again the following day. One day after “pre” (“post”), the mice were i.p. injected with DZP (1.0 mg/kg) or VEH (10 mL/kg) 30 min before the start of the three-compartment conflict task (Fig. 4a). Effective doses of DZP were selected based on the literature^32,33^. Criterion B consisted of three conditions to avoid the floor and ceiling effects: first, the average SAL for all sucrose trials was greater than 30 s; second, the latency for at least three sucrose trials was greater than 30 s; finally, the latency for all sucrose trials was less than 5 min. It took 2.58 ± 0.26 days for the mice to reach criterion B. The weight of the mice was maintained within ± 2% between the “pre” and “post” phases. Data from the fourth, fifth, seventh, eighth, and tenth trials of the conflict tests were used. The SAL, FR, and TSC relative to those of the “pre” are shown in Fig. 4b–d. For NOR, the differences between “post” and “pre” are shown in Fig. 4e and f, because the ratio cannot be calculated when the NOR of “pre” is 0.

### Data analysis

All values are reported as the mean ± standard error of the mean. Data were analysed using the paired *t*-test, Student’s *t*-test, Tukey’s multiple comparisons test after one-way analysis of variance (ANOVA), and Sidak’s or Dunnett’s multiple comparisons test after repeated measures ANOVA (RM-ANOVA) using GraphPad Prism 7 software (GraphPad Software, La Jolla, CA, USA). Differences were considered statistically significant at *P* < 0.05.

### Supplementary Information

Supplementary Information.

## Data Availability

The datasets generated during and/or analysed during the current study are available from the corresponding author upon reasonable request.
